# Adding High-Intensity Interval Training to Classical Resistance Training Does Not Impede the Recovery from Inactivity-Induced Leg Muscle Weakness

**DOI:** 10.3390/antiox12010016

**Published:** 2022-12-22

**Authors:** Tomas Venckunas, Marius Brazaitis, Audrius Snieckus, Mantas Mickevicius, Nerijus Eimantas, Andrejus Subocius, Dalia Mickeviciene, Håkan Westerblad, Sigitas Kamandulis

**Affiliations:** 1 Institute of Sports Science and Innovations, Lithuanian Sports University, 44221 Kaunas, Lithuania; 2 Kaunas Hospital of the Lithuanian University of Health Sciences, 50161 Kaunas, Lithuania; 3 Department of Physiology and Pharmacology, Karolinska Institutet, 171 77 Stockholm, Sweden

**Keywords:** inactivity, muscle weakness, muscle thickness, low-frequency force depression, resistance training, high-intensity interval training

## Abstract

Inactivity is known to induce muscle weakness, and chronically increased levels of reactive oxygen species (ROS) are proposed to have a central causative role in this process. Intriguingly, high-intensity interval training (HIIT), which involves bursts of high ROS production, can have positive effects in pathological conditions with chronically increased ROS. Here, young male volunteers were exposed to 3 weeks of unloading of the dominant leg followed by 3 weeks of resistance training without (Ctrl group) or with the addition of all-out cycling HIIT. Changes in muscle thickness were assessed by ultrasonography, and contractile function was studied by measuring the torque during maximal voluntary contractions (MVC). The results show an ~6% decrease in vastus lateralis thickness after the unloading period, which was fully restored after the subsequent training period in both the Ctrl and HIIT groups. MVC torque was decreased by ~11% after the unloading period and recovered fully during the subsequent training period in both groups. All-out cycling performance was improved by the 3 weeks of HIIT. In conclusion, the decline in muscle size and function after 3 weeks of unloading was restored by 3 weeks of resistance training regardless of whether it was combined with HIIT.

## 1. Introduction

Physical inactivity and skeletal muscle disuse are known to result in muscle weakness via a combination of muscle wasting and impaired contractility, i.e., decreased force production per cross-sectional area [1,2]. Inactivity-induced muscle atrophy has been linked to a sustained increase in the production of reactive oxygen species (ROS), and dysfunctional mitochondria are proposed to have a central role in the accelerated ROS emission [3,4,5]. Chronically increased ROS production has also been linked to impaired contractile function within the muscle fibers, and the resulting force production decrease is thus added to the reduction caused by a reduced muscle cross-sectional area [6,7].

Endurance-type exercise also increases ROS production, which in this case, is associated with beneficial effects and represents an integral trigger for training-induced adaptations [8,9]. In fact, the effect of both endurance and resistance training can be blunted by effective antioxidant treatment [10,11,12]. Intriguingly, high-intensity interval training (HIIT), which involves short periods of very high ROS production [13,14], induces beneficial effects on muscle function in pathological conditions with muscle weakness where chronically increased ROS is presumed to play a central causative role [15,16,17,18].

How to effectively design concurrent resistance and endurance training is a critical issue in the context of pathological conditions with impaired muscle function, as well as optimizing athletic performance [19,20]. The cellular signaling involved in triggering adaptations differs between these two training modalities, and the current knowledge about how these signaling pathways interact is limited [21,22,23]. Indeed, in some examples, adding endurance-type training to resistance training has negative as well as positive effects on overall muscular performance [24,25,26].

In the present study, we assess the recovery of muscle size and force production after three weeks of severe inactivity, including leg unloading [27]. During the three weeks following the unloading period, individuals performed resistance training with and without the addition of endurance training in the form of all-out cycling HIIT. Based on previous studies showing positive effects of bursts of increased ROS production in pathological conditions with chronically elevated ROS levels, we hypothesized that adding HIIT to resistance training would not impair the recovery of the inactivity-induced muscle weakness.

## 2. Materials and Methods

### 2.1. Volunteers and Interventions

Recreationally active young male volunteers participated in the study. All participants were healthy with no history of cognitive or neurological disorders, lower limb injury, or chronic disease such as diabetes, osteoarthritis, deep vein thrombosis, or cardiovascular disorders. At the onset of the study, participants were moderately active (2259 ± 299 MET min/week, mean ± SEM, *n* = 16) as assessed by the International Physical Activity Questionnaire (IPAQ) [28], and none of them were engaged in any structured training program or competitive sport. The Regional Biomedical Research Ethics Committee approved the study (no. BE-2-47). All participants provided written informed consent before participating. They were instructed to maintain their regular diet, and daily step counts were recorded in most participants during the unloading and training periods with a commercially available wrist-worn sleep/activity tracker (Xiaomi Mi band 2, China).

All participants underwent three weeks of unloading of the dominant leg, which was achieved by wearing a 10 cm extended-sole shoe on the non-dominant leg. In addition, they were instructed to move no more than a maximum of 2000 steps/day with the aid of forearm crutches [27]. Following the unloading and low-activity period, participants were randomly selected to perform three weeks of training consisting of either nine resistance training sessions (control, Ctrl) or the resistance training sessions plus eight HIIT sessions. Pre-intervention characteristics of the individuals in the two groups are shown in Table 1. Muscle volume and contractile function of the dominant leg knee extensor muscles were assessed at baseline, after the three weeks of unloading, and after the subsequent three weeks of training (48 h after last resistance training session). Measurements of vastus lateralis muscle thickness and 20 Hz electrically evoked torque were obtained in all individuals, whereas reliable measurements of maximum voluntary contraction (MVC) torque are missing for one individual in each group.

### 2.2. Training after Unloading Period

After the three weeks of dominant leg unloading, participants in the Ctrl group performed resistance training three times per week (on inconsecutive days) for three weeks. Training sessions were composed of three sets each of leg presses, knee extensions, and hamstring curls, all of which were performed using machine-based training equipment. These exercises were chosen as they are commonly included in strength-type resistance training programs and shown to effectively improve muscle size and function [29]. Each leg was trained separately, and each exercise started with the unloaded (dominant) leg. Sets for the dominant leg were carried out until failure with the resistance adjusted to reach failure within 8 to 12 repetitions. To achieve bilateral balance, the non-dominant leg was trained with identical absolute resistances and volumes to those of the dominant leg. The duration of the whole workout was ~40 min. All workouts were performed under supervision by a member of the research team.

Participants in the HIIT group performed three sessions per week of the same resistance training protocol as the Ctrl group. In addition, HIIT cycling exercise sessions were performed on days without resistance training. HIIT consisted of 30 s all-out cycling bouts at 0.75 Nm/kg body weight on a stationary ergometer bicycle; participants rested for 4 min between cycling bouts The number HIIT bouts in each session progressed from four (week 1), via five (week 2), to six (week 3).

### 2.3. Muscle Function

Electrically evoked and MVC isometric torques of the dominant leg knee extensors (m. quadriceps) were measured using an isokinetic dynamometer (System 3, Biodex Medical Systems, Shirley, MA, USA). The participants were placed in the dynamometer chair with the knee joint adjusted to an angle of 60° (full knee extension = 0°). To minimize changes in body position during contractions, the shank, trunk, and shoulders were tightly fastened by belts with Velcro straps. Transcutaneous muscle stimulation was applied via a pair of carbonized rubber electrodes covered on the inner surface with a thin layer of electrode gel (ECG–EEG Gel, Medigel, Modi’in, Israel). One electrode (6 cm × 11 cm) was placed transversely across the width of the proximal segment of the m. quadriceps close to the inguinal ligament, and the other electrode (6 cm × 20 cm) was placed above the distal segment of the muscle close to the patella. An electrical stimulator (MG 440, Medicor, Budapest, Hungary) was used to deliver 1 ms square-wave current pulses at a supramaximal amplitude, i.e., the voltage was 10% higher than that required to elicit peak torque in response to a single pulse. Maximum torques in response to 1 s trains of pulses given at 20 Hz and during MVCs were measured. A rest period of 1 min was set between contractions. Two MVCs were performed, during which participants received strong vocal encouragement to achieve maximal muscle activation. Each MVC was maintained for 3–4 s.

### 2.4. Ultrasonography

While the participant was lying on a massage table, transverse images of the vastus lateralis and intermedius mid-belly muscle thickness were obtained using B-mode ultrasonography with a 10–15 MHz transducer (Echoblaster 128, UAB; Telemed, Vilnius, Lithuania) and with minimal probe pressure application. The clearest images of the fascia captured were analyzed, with the probe placement outlined using a permanent marker at the middle point between the lateral epicondyle and greater trochanter of the dominant leg. The muscle thickness was assessed as the distance between the interfaces of adipose tissue–muscle and muscle–bone at the middle of the image (see Figure 3A). Three transverse images of muscle were obtained and then transferred to a computer for an assessment of muscle thickness using ImageJ image analysis software (Wayne Rasband, NIH, Bethesda, MD, USA). The average thickness of the three vastus lateralis images was used for further analyses.

### 2.5. Blood Lipid Peroxidation and Total Antioxidant Capacity

Blood samples from the antecubital vein of the non-dominant arm were collected from six participants in each group under resting conditions on three occasions: before starting the unloading period; after the three weeks of unloading; and after the subsequent three weeks of training (48 h after the last resistance training session). Blood samples were collected in 3 mL vacuum tubes containing the anticoagulant EDTA-K3 (Beckton-Dickinson, San Jose, CA, USA). The tubes were slowly inverted 5–10 times and plasma was thereafter separated by centrifuging at 2000× *g* for 15 min at 4 °C. The plasma was immediately aliquoted into 1.5 mL microtubes (FL Medical, Italy) and stored at –80 °C.

Oxidative stress was assessed by using an assay kit (MAK085, Sigma-Aldrich, St. Louis, MO, USA), which determines the plasma level of malondialdehyde (MDA), an end product of lipid peroxidation, via the reaction of MDA with thiobarbituric acid (TBA) to form a colorimetric product in proportion with the MDA present [30]. In this assessment, 20 µL of plasma was gently mixed with 500 µL of 42 nM sulfuric acid in a microcentrifuge tube. Next, 125 µL of phosphotungstic acid solution was added, and the solutions were mixed by vortexing. The microcentrifuge tube was then left at room temperature for 5 min before being centrifuged at 13,000× *g* for 3 min. The supernatant was discarded, and the pellet was transferred to a tube containing 2 µL of butylated hydroxytoluene (BHT, 100×) in 100 µL of purified water on ice, and additional purified water was added to adjust the volume to 200 µL. After that, 600 µL of TBA solution was added, and the solution was heated at 95 °C for 60 min followed by cooling on ice for 10 min. Next, 300 µL of 1-butanol and 100 µL of 5 M NaCl were added, and tubes were vortexed and centrifuged for 3 min at 16,000× *g* at room temperature. The 1-butanol layer was transferred to another tube, and 1-butanol was evaporated by heating at 55 °C. Finally, 200 µL of ultrapure water was added, and after mixing, the absorbance at 532 nm was measured and calibrated against an MDA concentration standard curve [30].

The total antioxidant capacity (TAC) of small molecules was assessed with an assay kit (MAK187, Sigma-Aldrich) where the absorbance of a colorimetric probe, formed in proportion to the ability to reduce Cu^2+^ to Cu^+^, is measured. Plasma was diluted 1:1 with 5 µL of a Protein Mask, and purified water was added to a final volume of 100 µL. Then, 100 µL of Trolox Standard was added, and samples were incubated with 100 µL of Cu^2+^ reagent solution for 90 min at room temperature, before measuring the absorbance at 570 nm. The absorbance was calibrated against 0–20 nmol/µL Trolox Standard solutions. Thus, the antioxidant capacity is expressed as Trolox equivalents.

All samples and standards for calibrations were run in duplicate on a Spark multimode microplate reader (Tecan, Grödig, Austria), and the average of the two values was used in subesquent calculations.

### 2.6. Statistical Analysis

Statistical analyses were performed with Sigmaplot 13 (Systat Software Inc., San Jose, CA, USA). The Shapiro–Wilk test was used to confirm that data were normally distributed. Two-way repeated measures analysis of variance (two-way RM ANOVA) was used to isolate statistically significant effects in relation to time (pre, after unloading, and after training) and differences between the Ctrl and HIIT groups; this was followed by the Holm–Sidak post hoc test, in which significant differences were detected. One-way RM ANOVA with the Holm–Sidak post hoc test was used to assess changes in power output during HIIT cycling. Significance was set at *p* < 0.05. Data are presented as individual values (torques, muscle thickness, and power output) and mean ± SEM.

## 3. Results

Participants were severely inactive during the 3-week unloading period compared to the subsequent 3-week training period with step counts amounting to 1003 ± 182 and 6135 ± 1024, respectively (*p* < 0.001, *n* = 11).

**Figure 1:**
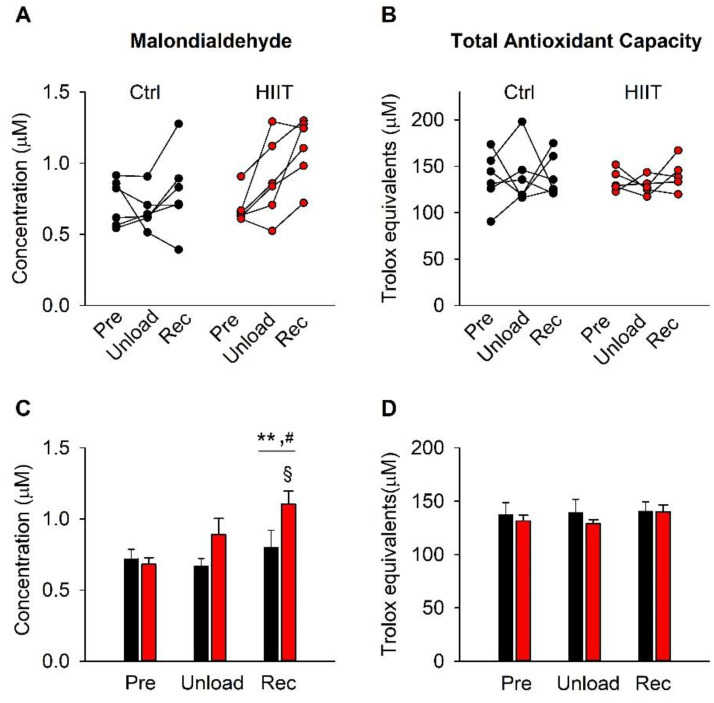
Malondialdehyde (MDA) concentration and total antioxidant capacity (TAC) at the individual level (**A**,**B**) and mean ± SEM data (**C**,**D**) obtained before interventions (Pre), after the 3-week unloading period (Unload), and after the subsequent training period (Rec). Ctrl, black bars and symbols; HIIT, red bars and symbols. Two-way RM ANOVA with Holm–Sidak post hoc test: difference between Ctrl and HIIT, § *p* < 0.05; overall time effect Rec vs. Pre, ** *p* < 0.01; overall time effect Rec vs. Unload, ^#^ *p* < 0.05.

We measured plasma MDA and TAC to get an overall view of the extent of ROS challenge during the inactivity period and the subsequent training periods (Figure 1). The initial three weeks of inactivity were the same for the Ctrl and the HIIT groups. During this period, the average MDA level showed a small (~10%) increase, which did not reach statistical significance (*p* = 0.23). Conversely, the average MDA concentration showed an increase during the following training period. Moreover, the MDA concentration was significantly higher (*p* = 0.021) in the HIIT than in the Ctrl group at the end of the training period (Figure 1A,C).

We observed no consistent difference in TAC over time or between groups (Figure 1B,D). 

**Figure 2:**
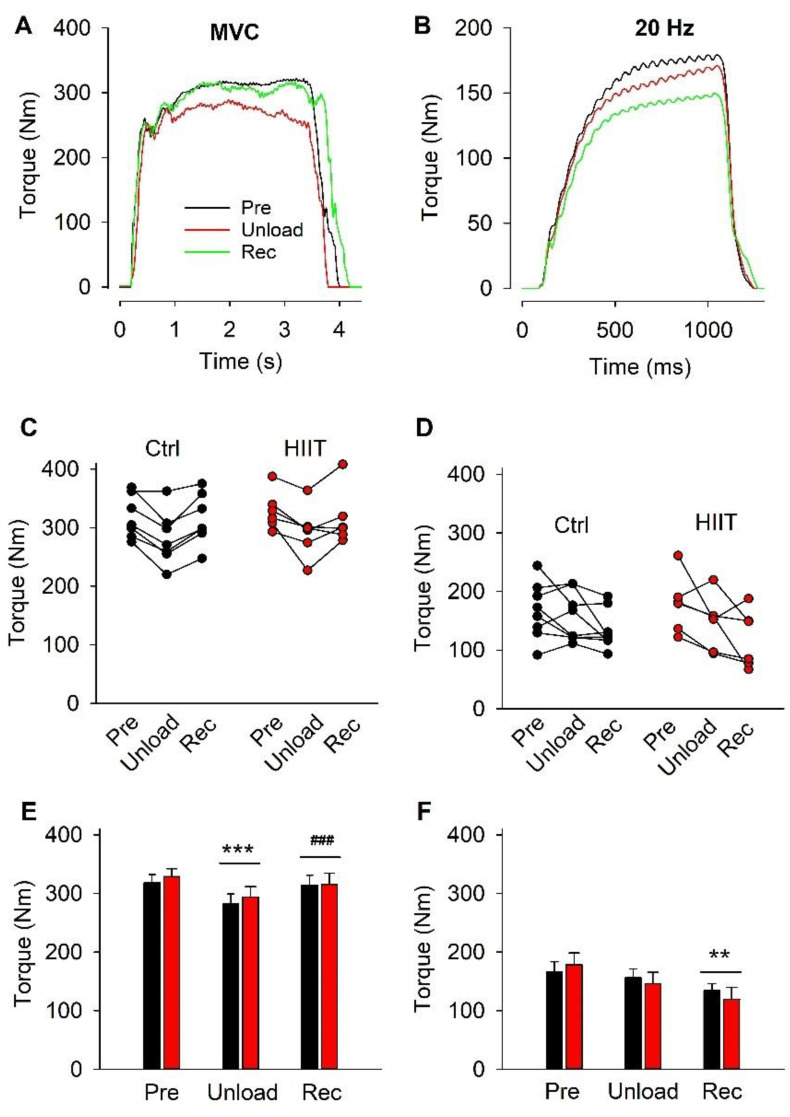
Representative torque records obtained for a participant in the Ctrl group during MVC (**A**) and 20 Hz contractions (**B**) produced before interventions (Pre, black line), after the 3-week unloading period (Unload, red line), and after the subsequent training period (Rec, green line). MVC and 20 Hz torques at the individual level (**C**,**D**) and mean ± SEM data (**E**,**F**). Ctrl, black bars and symbols; HIIT, red bars and symbols. Two-way RM ANOVA with Holm–Sidak post hoc test: no statistically significant difference between the two groups was found at any time; overall time effect Unload or Rec vs. Pre, ** *p* < 0.01 and *** *p* < 0.001, ^###^ *p* < 0.001; overall time effect Rec vs. Unload.

Torques during MVC and 20 Hz stimulated contractions were measured before and immediately after the unloading period as well as after the subsequent training period. Representative MVC and 20 Hz torque records obtained from a participant in the Ctrl group are shown in Figure 2A,B. Note that the MVC torque was lower after the unloading period than on the other two occasions, whereas the lowest 20 Hz torque was observed after the training period. Data at the individual level and mean data show similar patterns in the Ctrl and HIIT groups (Figure 2C–F). Thus, MVC torque was significantly decreased by about 11% after the unloading period and fully restored after the subsequent training period, whereas the 20 Hz torque was only significantly decreased after the training period. Importantly, there was no significant difference between the two groups in any instance.

**Figure 3:**
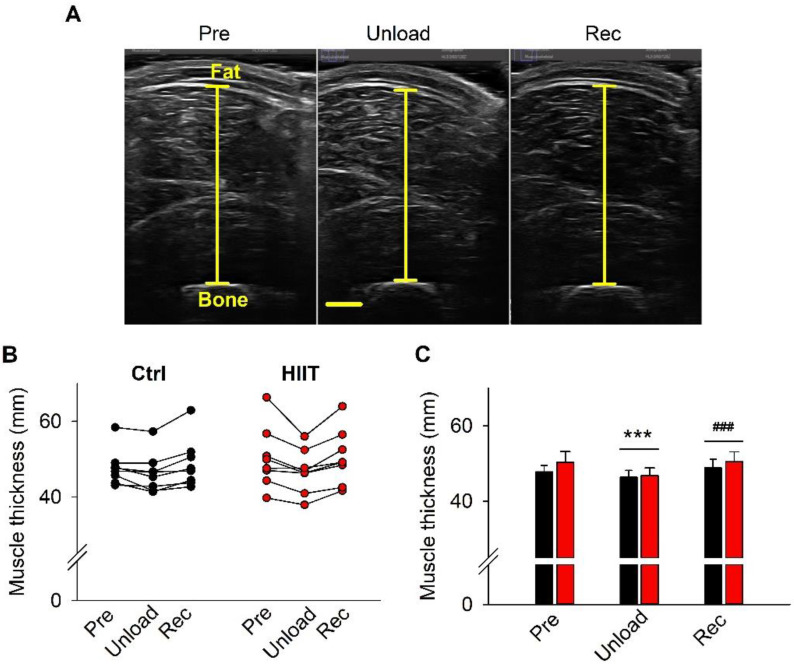
(**A**) Representative ultrasonographic images of vastus lateralis and intermedius. Muscle thickness was measured as the distance from the subcutaneous fat to the bone as indicated. Scale bar in Unload equals 10 mm and applies to all three panels. Individual data points (**B**) and mean ± SEM (**C**) muscle thickness. Ctrl, black bars and symbols; HIIT, red bars and symbols. Two-way RM ANOVA with Holm–Sidak post hoc test: no difference between the two groups was found at any time; overall time effect Unload vs. Pre, *** *p* < 0.001; overall time effect Rec vs. Unload, ^###^ *p* < 0.001.

Figure 3A shows representative ultrasonographic images used to measure vastus lateralis and intermedius muscle thickness. Individual and mean data from these measurements show a significantly decreased muscle thickness (~6%) after the 3-week inactivity period, which was fully restored during the subsequent 3-week training period and with no difference between the Ctrl and HIIT groups at any time (Figure 3B,C).

**Figure 4:**
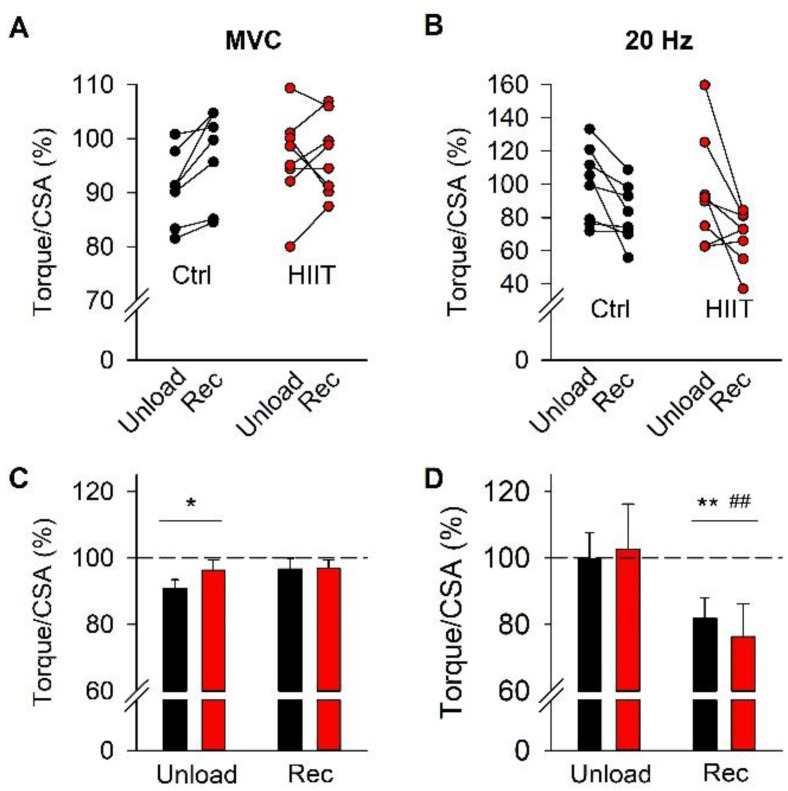
Individual data and mean (± SEM) of relative MVC torque (**A**,**C**) and 20 Hz torque (**B**,**D**). Data are presented as the percentages of Pre values for each individual. Ctrl, black bars and symbols; HIIT, red bars and symbols. Two-way RM ANOVA with Holm–Sidak post hoc test; no difference between the two groups was found at any time; overall time effect Unload or Rec vs. Pre, * *p* < 0.05 and ** *p* < 0.01; overall time effect Rec vs. Unload, ^##^ *p* < 0.01.

Changes in absolute torque during interventions (see Figure 2) can, in principle, be due to changes in either muscle CSA or contractile function within muscle fibers. To distinguish between these two possibilities, we compared changes in torque vs. muscle thickness with values obtained before the interventions, set to 100% in each participant (Figure 4). A significant reduction in MVC torque after the unloading period was observed even after taking the reduction in muscle thickness into account (Figure 4A,C), although the relative decrease was smaller (~6%) than the decrease in absolute MVC torque (~11%). The relative torque in 20 Hz contractions was not affected by the unloading period, whereas it was decreased by about 22% after the subsequent training period (Figure 4B,D), which was similar to the decrease in absolute 20 Hz torque (~23%). Notably, the relative MVC and 20 Hz torques showed no statistically significant difference between the Ctrl and HIIT groups at any time point. 

**Figure 5:**
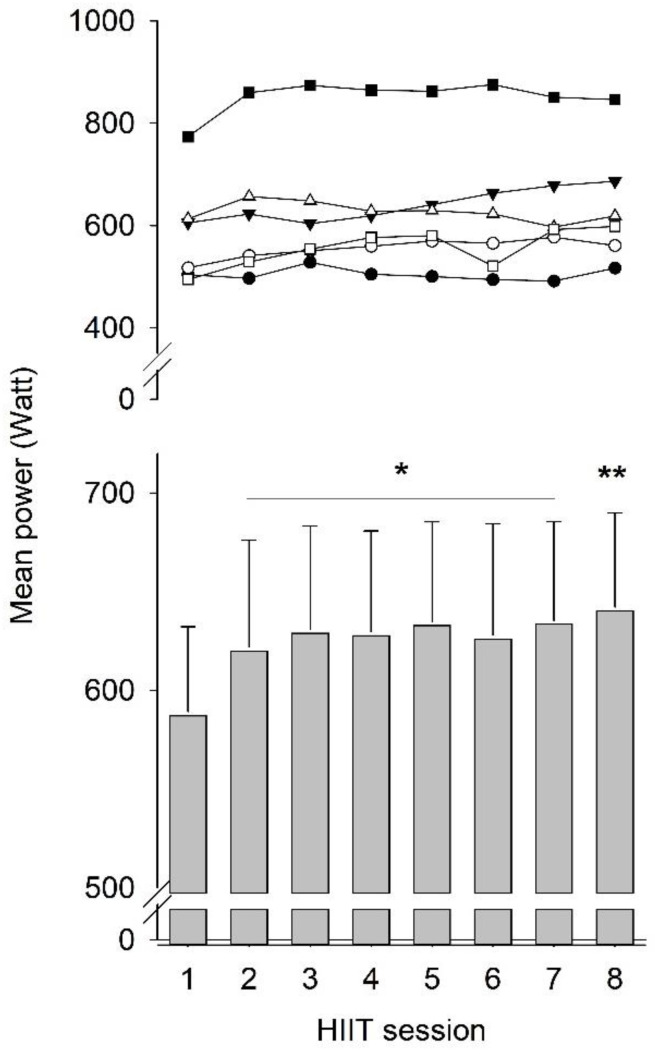
Individual data (upper row) and mean (±SEM; lower row) of average cycling power during the eight HIIT sessions. * *p* < 0.05 and ** *p* < 0.01 vs. first HIIT session with one-way RM ANOVA followed by Holm–Sidak post hoc test.

To assess the effect of HIIT on performance, we measured the power output during cycling bouts in each session for six out of the eight individuals performing HIIT. The HIIT protocol involved a gradual increase in cycling bouts during sessions from four bouts during the first week, via five bouts during the second week, to six bouts during the third week. To obtain comparable data, we calculated the mean power output during the initial four cycling bouts of each session. Compared to the first HIIT session, the results show a marked (5.1 ± 1.7%) increase in power output already in the second session, which was followed by a slower improvement, reaching an increase of 9.1 ± 3.0% in the last session (Figure 5). 

## 4. Discussion

In this study, we have showed that three weeks of inactivity and leg unloading resulted in an ~10% decrease in MVC torque, which can be attributed to the combined effect of muscle atrophy and impaired contractile function intrinsic to the muscle fibers. This muscle weakness was fully restored after three weeks of training. Importantly, recovery of muscle size and MVC torque-generating capacity was not impaired in the participants where endurance-type training in the form HIIT was added to the resistance training performed by all participants; notably, these recovery similarities occurred despite an increased ROS challenge, as evidenced by a higher plasma MDA concentration, in the HIIT group.

We used ultrasonographic measurements of the vastus lateralis and intermedius muscle thickness to assess the extent of inactivity-induced muscle atrophy and the subsequent recovery. Although this method does not allow a direct measurement of the muscle cross-sectional area, previous studies have shown a good correlation between changes in vastus lateralis muscle thickness, as measured by ultrasound, and the anatomical cross-sectional area, as measured with magnetic resonance imaging, both over time under control conditions and after a period of resistance training [31,32]. The ultrasound measurements showed an ~6% decrease in muscle thickness during the inactivity period, which implies a similar decrease in cross-sectional area that can account for half of the 11% decline in MVC torque, and the remaining deficit would then be due to reduced force-generating capacity of the cellular contractile machinery. Although we did not detect any significant increase in plasma MDA after the inactivity period, there is substantial experimental support for increased ROS production during muscle disuse [33,34,35,36], which can be linked to both muscle atrophy and impaired muscle fiber contractility [5].

The recovery of force at low stimulation frequencies can be slow, and this prolonged low-frequency force depression (PLFFD) has been observed after both mechanically and metabolically demanding exercise [37,38,39]. Our results showed a decrease in 20 Hz torque at the end of the 3-week training period, which indicated that the 48 h of rest after the last training session was not long enough to allow full recovery from PLFFD. Interestingly, the extent of PLFFD did not differ between the two groups. Thus, the addition of HIIT to the resistance training did not exaggerate PLFFD, which indicates that the PLFFD was mainly due to mechanical stress-induced myofibrillar dysfunction [40].

The recovery of muscle thickness and contractile function after the inactivity period was not negatively affected by the addition of HIIT, which resulted in an increased ROS challenge, as evidenced by higher plasma MDA in the HIIT than in the Ctrl group. From this perspective, it appears safe to add bursts of high ROS production during HIIT to the inactivity-induced muscle disturbances, where a prolonged increase in baseline ROS production has an important causative role [3,4,5]. Moreover, the cycling performance during HIIT sessions was improved during the 3-week training period. A significant increase in power output was observed already in the second HIIT session, which would be too early for improved muscular endurance to occur and is therefore most likely to reflect an enhanced neuronal muscle activation during the all-out cycling bouts [41]. After this initial rapid increase, cycling performance showed a further improvement that might involve adaptations toward improved energy metabolism and muscular endurance [15,42,43].

In conclusion, muscle size and force from inactivity-induced muscle weakness can be successfully regained within three weeks of resistance training. Adding HIIT with bursts of increased ROS production did not have any negative impact on force recovery and improved all-out cycling performance, which adds support to previous studies showing positive effects of HIIT in pathological conditions with ROS-related muscle weakness [44,45].

## Figures and Tables

**Table 1:** Characteristics of the participants (mean ± SEM).

	Ctrl Group (*n* = 8)	HIIT Group (*n* = 8)
Age (years)	25.0 ± 1.6	26.9 ± 2.2
Height (cm)	180.9 ± 1.9	184.5 ± 3.2
Body mass (kg)	81.9 ± 3.5	84.4 ± 4.7

## Data Availability

Not applicable.
